# Qualitative study of carbides in liquid phase sintered M3:2 high speed steel

**DOI:** 10.1016/j.heliyon.2024.e33925

**Published:** 2024-07-01

**Authors:** Walid Khraisat, Wisam Abu Jadayil

**Affiliations:** a Industrial Engineering Department, The University of Jordan, Amman, 11942, Jordan; b College of Engineering, Abu Dhabi University, Abu Dhabi, 59911, United Arab Emirates

**Keywords:** High speed steel, Primary carbides, Eutectic carbides, Microstructure, Carbide-forming elements

## Abstract

The microstructure of liquid phase sintered M3:2 high speed steel and the effect of adding carbon and silicon on the microstructure was characterized by scanning electron microscopy, dispersive spectrometry, and X-ray diffraction. Various types of carbides were formed depending on the added carbon and/or silicon, the sintering atmosphere and the cooling rate. The microstructure of sintered M3:2 high speed steel samples in vacuum conditions without the addition of Si and graphite is composed mainly of MC and M_6_C carbides inside cells and primarily at cell boundaries. The M_2_C eutectic carbides in these samples were formed at cell boundaries and their amounts and morphology depends on the cooling rate. Sintered samples in N_2_ atmosphere with added carbon and silicon, M_6_C eutectic and M_7_C_3_ eutectic carbides were dominantly formed while carbonitrides *were formed in smaller amounts.*

## Highlights

• The microstructure of liquid phase sintered M3:2 high speed steel has various types of carbides were formed depending on the added carbon and/or silicon, sintering atmosphere and the cooling rate.
• The M2C eutectic carbides in these samples were formed at cell boundaries and their amounts and morphology depends on the cooling rate.
• Sintered samples in N2 atmosphere with added carbon and silicon, M6C eutectic and M7C3 eutectic carbides were dominantly formed while carbonitrides were formed in smaller amounts.

## 1 Introduction and literature review

High speed steels (HSSs) are complex iron-base alloys with high content of C (0.5 %–1.65 %) and carbide forming elements like Mo, W, Cr and V [1,2]. The microstructure and properties of the HSS are due to the synergic effect of these alloying elements and heat treatment. The microstructure of HSS consists of a martensitic matrix with a dispersion of three types of high hardness and wear resistant carbides. These carbides are called primary, eutectic and secondary carbides. Primary and eutectic carbides solidify directly from the melt. Eutectic carbides which are coarse and unevenly distributed are formed due to the segregation of the alloying elements during solidification of the liquid phase [3,4]. These eutectic carbides have deteriorating effects on the mechanical properties [5]. Therefore, the control of the morphology, size and amount of these eutectic carbides is an important precondition for improving the properties of HSS.

The main types of carbides found in high-speed steels are the MC carbides (Nb and V-rich) which can be found as large blocky particles termed primary carbides because they form directly from the melt. The MC carbide has a higher V level and lower Fe and Cr levels in comparison to other carbides as M_6_C and M_2_C. The M_6_C carbide is rich in the heavy elements Mo and W which are normally present in M_6_C at the same level as they are in total composition in the HSS [5]. The formation of M_6_C is favored by high levels of C and Mo. For a given amount of V, increasing the amount of W to higher levels favors the formation of M_6_C at the expense of MC type whereas a high amount of Mo has the opposite effect [6]. The addition of Si to HSS favors the formation of pearlite in the matrix, suppressing the formation of martensite in the matrix and the formation of grain boundary carbides [7,8]. The main compositional difference between MC, M_6_C and M_2_C carbides is that M_6_C contains the highest Fe and MC contains the lowest Fe content [[9], [10], [11]].

The M_2_C carbide (Mo/W-rich) forms during rapid cooling or at high carbon content. The morphology of M_2_C can be classified into two types, namely the lamellar shape and the rod-like shape [7,8]. Lamellar-like morphology is formed at low cooling rates or high V content while rod-like morphology is favored by high cooling rates or minor contents of N and Ca [10,11]. The Cr rich M_7_C_3_ carbide is a metastable carbide and is mainly distributed along grain boundaries [11].

The MC, M_2_C, M_6_C, and M_7_C_3_ carbides can be solidified as primary and/or eutectic carbides directly from the melt [12,13]. During solidification the liquid phase solidifies through different eutectic reactions leading to the formation of up to four eutectics: (γ + MC), (γ + M_6_C) or (γ + M_2_C) and (γ + M_7_C_3_) [14]. The morphology, size, distribution, and composition of these carbides are affected by the cooling rate, sintering atmosphere and the addition of alloying elements.

The present work aims to identify and characterize the different carbides present in liquid phase sintered M3:2 HSS using different techniques also to study the effect of the sintering cycle and the effect of the alloying elements Si and C on the morphology and distribution of carbides in as sintered M3:2 high speed steel. The effect of alloying elements Si and graphite on HSS is to promote the formation of unstable carbides like M3C and M2C carbides after sintering. These unstable carbides when HSS is heat treated will form fine secondary carbides like MC, M2C, M_6_C that are beneficial to the mechanical properties.

## 2 Research experimental work

### 2.1 Material

The base powder used in the experiments was spherical gas-atomized M3:2(ASP 2023) grade HSS supplied by Erasteel Kloster AB, Söderfors, Sweden. The chemical composition of the M3:2 HSS powder is shown in Table 1 with a particle size range of 50–150 μm. These values are taken from Erasteel Kloster AB.

**Table 1:** Chemical composition (wt-%) of M3:2 powder and samples 1-4.

Sample	HSS	C	V	W	Mo	Cr	Si	Mn
Sample 1 and 2	M 3:2	1.28	3.1	6.4	5	4.2	0.6	0.28
Sample 3	M3:2 + 3%Si	1.28	3.1	6.4	5	4.2	3.6	0.28
Sample 4	M3:2 + 0.5 %C+3%Si	1.78	3.1	6.4	5	4.2	3.6	0.28

Loose powder mixtures were prepared from M3:2 as base powder, elemental Si and graphite. The elemental powders added were in the micrometer range. The different samples prepared are shown in Table 2. The sample compositions and the different thermal conditions are designed to study their effect on the resulting microstructure. Sintering was carried out in a pilot furnace under high vacuum (10^−5^ mbar) with a heating rate of 20 °C/min to 1000 °C followed by10 °C/min to the sintering temperature 1270 °C and 10 min holding time followed by subsequent cooling either furnace cooling where the furnace is shut down after 10 min at the sintering temperature or using a cooling rate of 5 °C/min. Samples 1–3 were sintered in vacuum and sample 4 was sintered in a tubular furnace under N_2_ gas flow. A fully dense material made of the M3:2 grade powder was achieved by sintering at around 1270 °C.

**Table 2:** The thermal parameters for sintering the four samples.

Sample	Sintering temperature/sintering time/cooling rate
1	1270 °C/10min, 5 °C/min
2	1270 °C/10min, f.c
3	1270 °C/10min, 5 °C/min
4	1270 °C/10min, f.c

S.T sintering temperature, H.t holding time, C.R cooling rate and f.c. furnace cooled.

### 2.2 Characterization and testing

**Fig. 1:**
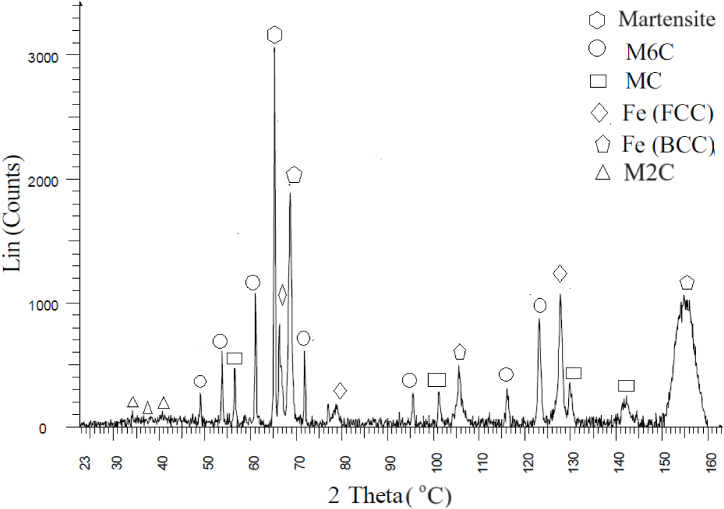
X-ray diffraction (XRD) pattern of sample 1.

**Fig. 2:**
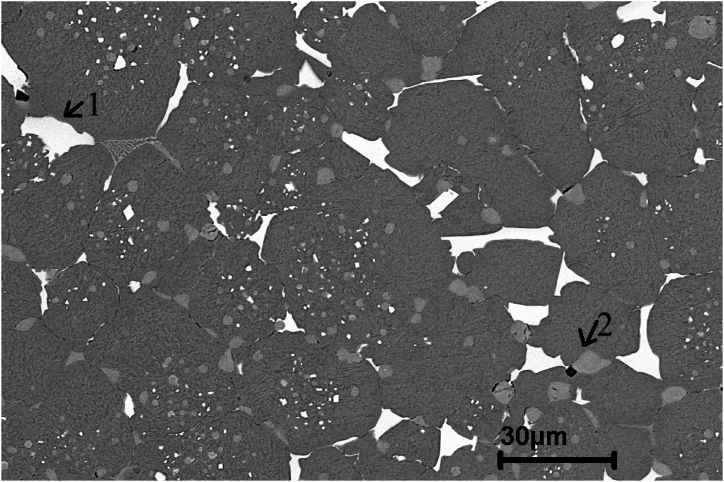
SEM micrograph of sample 1 showing martensitic matrix with M_6_C milky white contrast and MC along cell boundaries. Also can be seen one M_2_C eutectic colony (upper left).

To determine the type of carbide, present in the microstructure of as sintered M3:2 several identification techniques were used in this work as shown in Table 3. Besides the techniques mentioned in Table 3, X-ray diffraction was used (Bruker D8 Theta Advance instrument with Cr Kα radiation with a wavelength λ = 2.29 Å and with a scanning range of 20–160°) on sample 1, to decide whether or not M_2_C carbide is present in the as sintered material. The results are shown in Fig. 1. The obtained diffraction signals indicate clearly the presence of MC, M_6_C and M_2_C carbides. The intensity coming from M_2_C carbides is weak indicating a low content of M_2_C in sample 1. This is also confirmed by the microstructure of sample 1 which clearly shows M_2_C as seen in Fig. 2.

**Table 3:** Carbide identification methods.

Carbide	Morphology		Contrast under SE/BSE imaging mode
	Primary	Eutectic	
M_6_C	Polygonal shape	fish bone	milky white
M_2_C		-lamellar -rod-like	white to light grey
MC	Polyhedral, globular	Poorly coupled lamellar	grey
M_7_C_3_		-rod-like -blade-like	dark grey
M(C,N) Carbo Nitrides	Polygonal		black
MnS	Globular		grey

Density in the sintered compacts was measured using the Archimedes principle by weighing the samples dry, immersed and in wet conditions. The theoretical density was calculated by adding the phase constituents.

### 2.3 X-ray microanalysis (EDS)

**Fig. 3:**
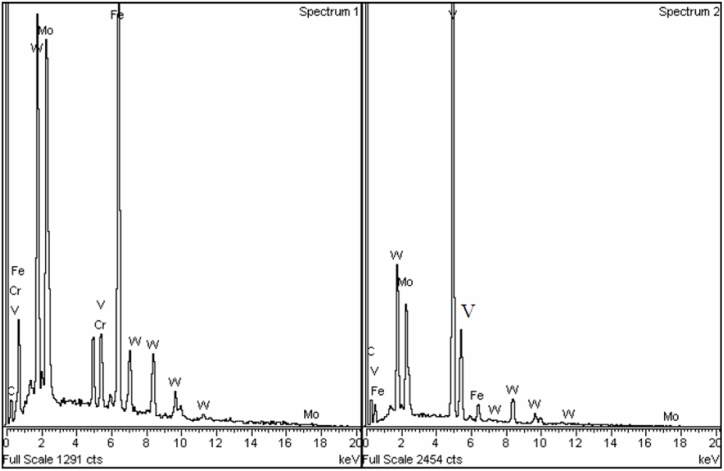
EDS spectra taken at 20 kV in points 1 and 2, respectively, in Fig. 2.

**Fig. 4:**
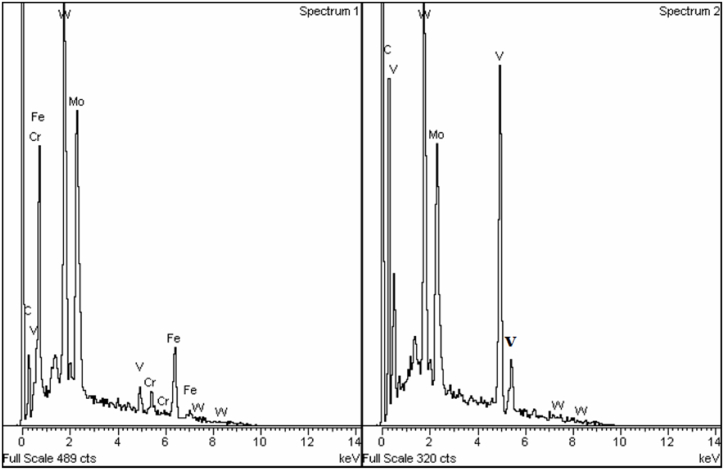
EDS spectra taken at 10 kV in points 1 and 2, respectively, in Fig. 2.

The metallographic samples were analyzed by OM and SEM equipped with EDS, after grinding, polishing and etching with Nital (6 % nitric acid alcohol). The chemical composition of the elements was determined by energy-dispersive X-ray spectroscopy (EDS) analysis using a LEO 1550 Germini instrument. Knowing that the X-ray signals come from a volume larger than the individual carbides, three levels of accelerating voltages were used (20, 15 and 10 kV) to decrease the signals coming from the bulk material. The M_6_C carbide is quite rich in Fe compared to MC, which is low in Fe and Cr but rich in V. EDS analysis was done at two locations point 1 and point 2 of sample 1 as shown in Fig. 3, Fig. 4.

The different carbides were analyzed using SEM images BSE mode and SE mode. Images of carbides contain different contrasts, carbides containing elements with higher atomic numbers (Z) appear brighter and dark carbides have lower Z. Carbides that contain elements with an atomic number lower than Fe like MC carbides (V-rich) appear darker than the matrix. However, carbides that contain elements with higher atomic numbers than Fe like M_6_C (Mo rich) carbides appear brighter than the matrix. Also the morphology of carbides was examined using Murakami etchant (3 g K3Fe (CN)6 + 10 g NaOH+100 ml H2O), in which M2C were selectively etched but not the matrix and MC carbides.

**Fig. 5:**
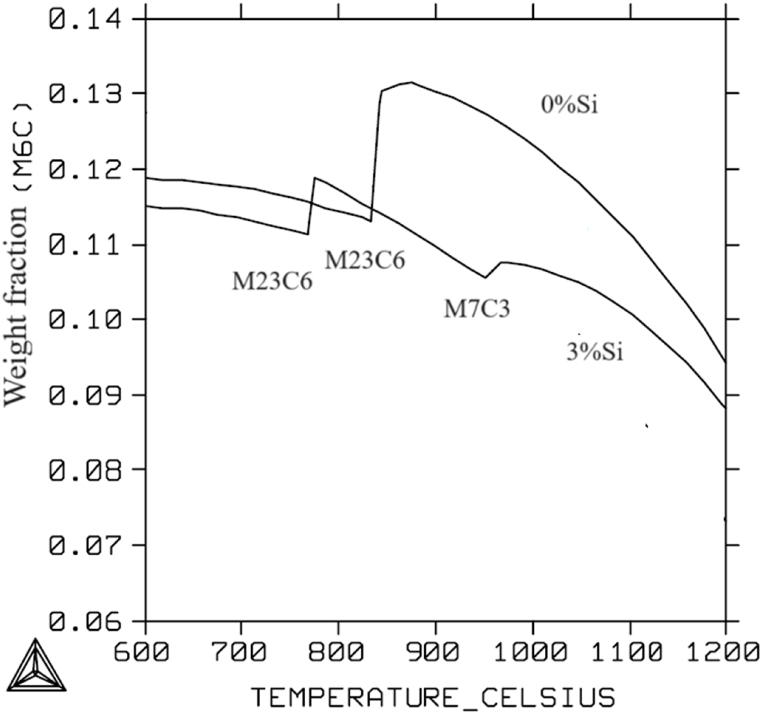
Weight fraction of the M_6_C carbide at different temperatures and at different wt% Si.

By using the program Thermo-Calc the theoretical amount of M6C carbide, at different temperatures and at different Si content was predicted using the database TCFE of the software. From Fig. 5 it can be seen that the weight fraction of M6C decreases with the addition of Si. Furthermore, as temperature decreases there is a sharp decrease in the amount of M6C at about 850 °C, 768 °C and 955 °C. This decrease can be attributed to the formation of other types of carbides like M23C6 and M7C3.

## 3 Results and discussion

**Fig. 6:**
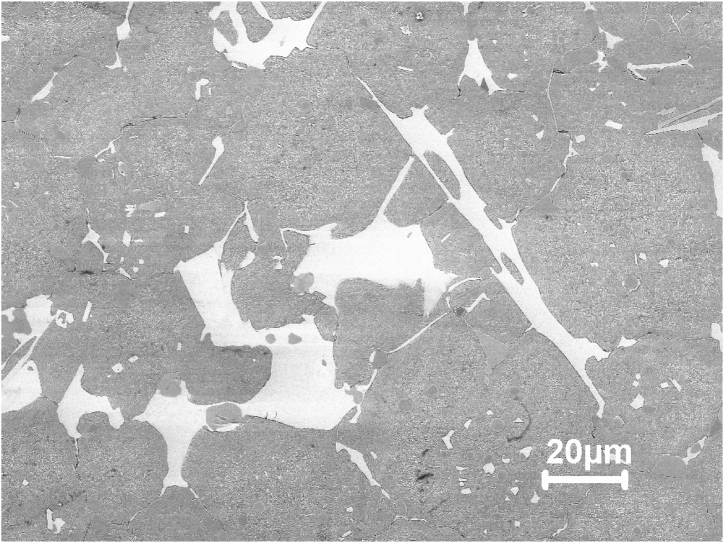
SEM micrograph of as sintered sample 1 showing martensitic matrix with M_6_C milky white carbide and MC grey carbides along cell boundaries.

Fig. 2, Fig. 6 are SEM micrographs of the microstructure of sample 1. The microstructures consist of martensite matrix and carbides that are mainly distributed at the martensite lath and at the grain boundaries. Fig. 3, Fig. 4 show the EDS spectra of points 1 and 2 depicted in Fig. 2. From the EDS analysis at point 1 comparing the Fe Lα peaks from spectrum 1 (20 kV) with spectrum 2 (10 kV) as shown in Fig. 3, Fig. 4 the Fe Lα peak is sharply increased to more than half the full scale, whereas the peaks Fe Kα1 and Fe Kα2 are strongly decreased. This means that the milky white carbide is rich in Fe. By doing the same comparison concerning the Fe signal for the grey carbide at point 2, we can notice the disappearance of Fe Lα leading us to conclude that this type of carbide is poor in Fe. Also, point 1 and point 2 showed intense W and Mo peaks and point 2 showed intense V peaks. The two Carbides exhibited two different contrasts under SEM/SE mode leading to the conclusion that point 1 corresponds to M_6_C carbide and point 2 corresponds to MC carbide. Eutectic carbides are not present in high amounts in sample 1 however there are some M_2_C carbides present in sample 1 as shown in Fig. 2 having a rod-like (fibrous) morphology.

The XRD patterns of sample 1 shown in Fig. 1 confirm the existence of MC, M_6_C, M_2_C carbides. It illustrates: i) the existence of the main carbides M_6_C and MC as well as the existence of ferrite, austenite, and martensite, ii) the high intensity for the main martensite (200) peak in sample 1 which is attributed to the fact that martensite is the dominant phase in the microstructure. It should be noted that the intensity of the Fe-fcc peaks at 66.9° (2ϴ) and 128.75° are affected by a MC peak at 66.7° as well as peaks for M_6_C and MC close to the Fe-fcc peak at 128.75°.

The intensity of the peaks of M_2_C carbide in sample 1 is very low which is directly proportional to the M_2_C amount present in the microstructure.

**Fig. 7:**
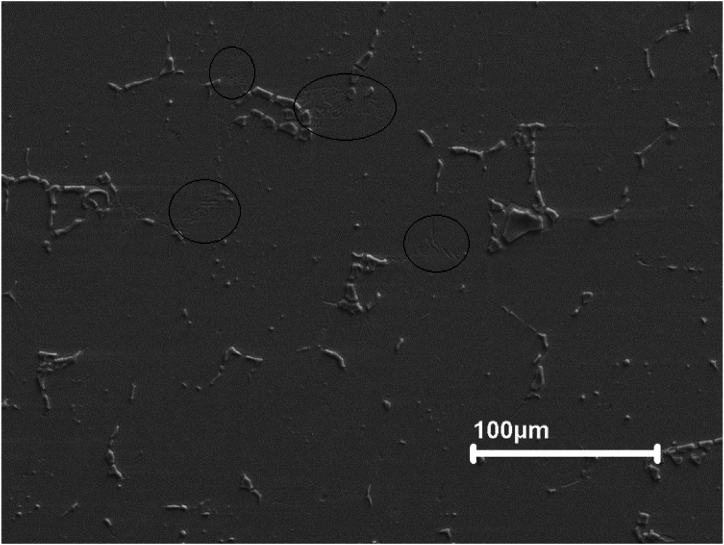
Microstructure of sintered M2 high-speed steel etched with Murakami's etchant at room temperature for long periods showing the MC carbides protruded from the matrix and the M2C carbides indented in the matrix (upper left).

Fig. 7 shows the microstructure of sample 1 after polishing and etching using Murakami's Etching solution at room temperature for longer periods of more than 5 min. The Murakami solution etched selectively the matrix and M_2_C carbides in sample 1 and completely etched out the M_2_C carbide. The MC carbides protruded from the matrix and the M_2_C carbides indented in the matrix.

**Fig. 8:**
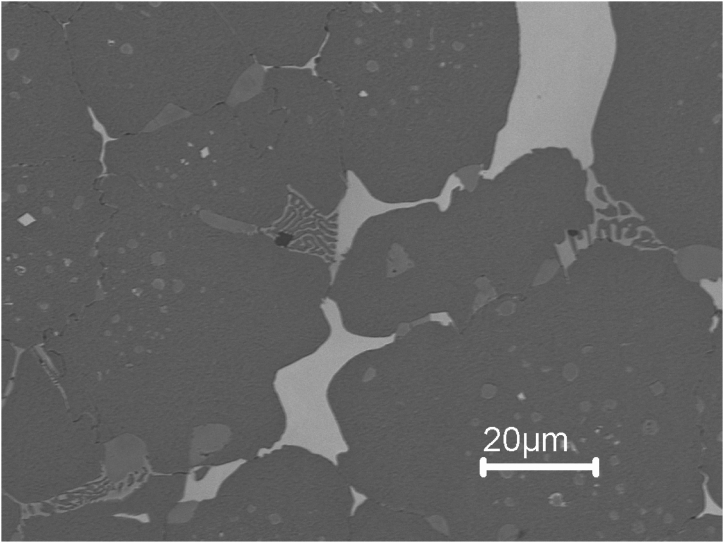
SEM micrograph of sample 2 showing a cluster of M_2_C rod-like morphology (light grey) and MC carbide (grey) and M_6_C (milky white). Also, MnS inclusion is seen located at M_2_C carbide in the middle of the image (dark grey).

**Fig. 9:**
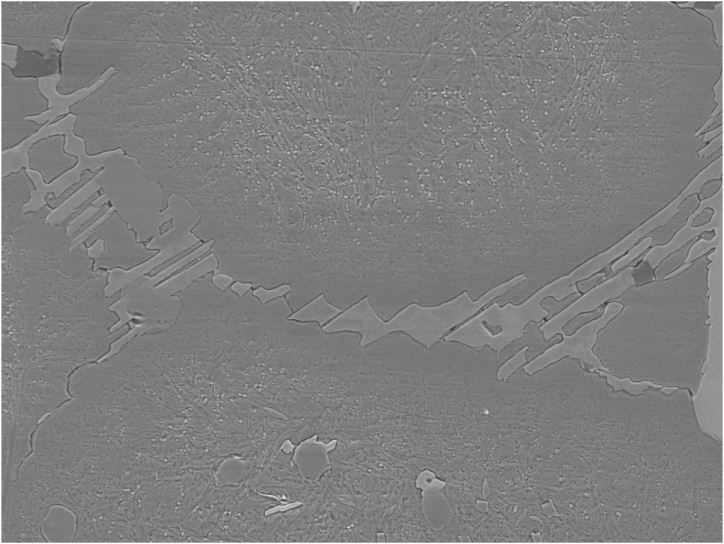
SEM micrograph of sample 2 showing a cluster of M_2_C lamellar morphology (light grey) and MC carbide (grey). Also evident in the microstructure MnS (dark grey) at the carbide/matrix interface.

However, in sample 2 the M_2_C carbides are present in higher amounts compared to sample 1 and the M_2_C carbide are present in the microstructure of sample 2 having a rod-like morphology as seen in Fig. 8 and a lamellar morphology as seen in Fig. 9. Examining the microstructures shown in Fig. 8, Fig. 9 one can observe that MC carbide is in the interior of the lamellar M_2_C and in the case of the rod-like morphology MC carbide is located at the M_2_C/matrix interface.

**Fig. 10:**
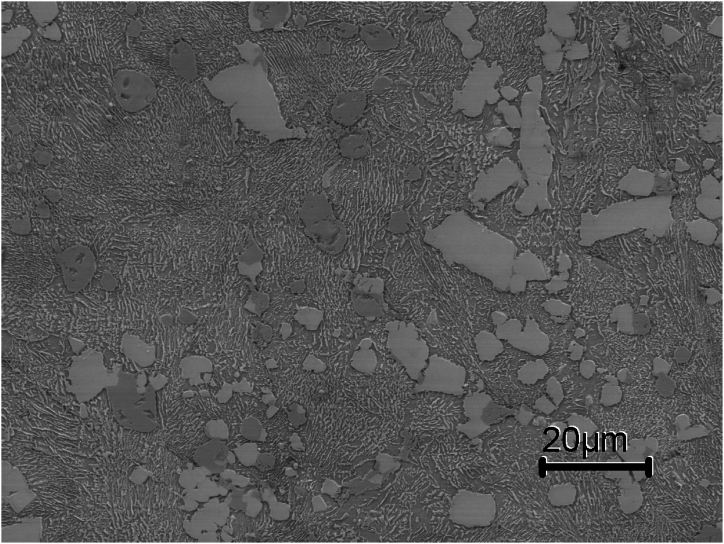
SEM micrograph of sample 3 showing a pearlitic matrix, MC (grey) and M_6_C(white) primary carbides. No eutectic carbides are present in sample 3.

**Fig. 11:**
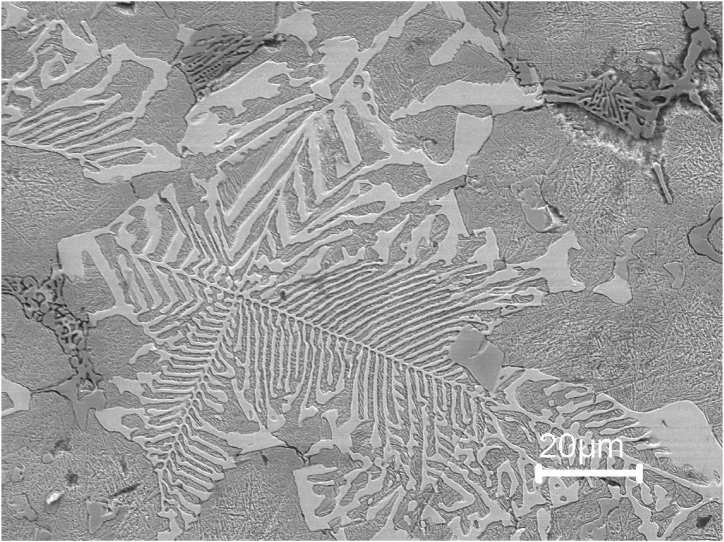
SEM micrograph of sample 4 showing martensitic matrix and three eutectic carbides. The fish bone morphology (milky white), rod-like colony(left-middle) and blade-like colony (upper right and upper left).

**Fig. 12:**
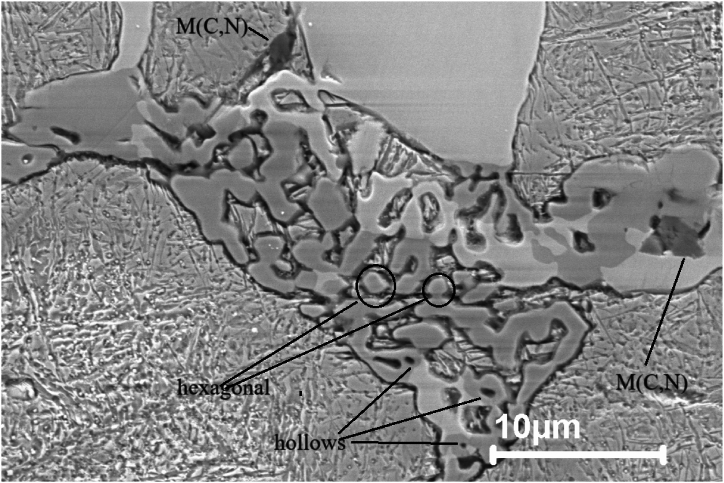
SEM micrograph showing the rod-like morphology of eutectic M_7_C_3_ carbide of sample 4.

Examining Fig. 8, Fig. 9, Fig. 10, Fig. 11, Fig. 12 compound carbides (mixed carbide cluster) of MC, M_2_C and M_6_C are present at grain boundaries. This means that these carbides are not independent of each other, because the solute atoms which are rejected by one carbide are usually needed for the growth of the other. This can be explained by the fact that with the formation of MC carbides, V is consumed from the residual liquid while elements like Mo (the lowest solubility at MC carbides) and Cr contents increase in the residual liquid. Meanwhile, the eutectic M_6_C carbides need plenty of alloying elements like Mo and W during precipitation. This will consume high amounts of V, Mo and W elements while enriching the liquid phase with Cr.

The importance of the alloying elements in high-speed steel and the thermal parameters like temperature and cooling rate result is the effect they have on the type of carbides formed with different crystal structures, morphology, and chemistry. Adding 3 wt% Si and using a cooling rate of 5 °C/min promoted the formation of M_3_C carbides in the HSS grains which is not predicted by thermodynamics as seen in Fig. 5. Also adding 3 wt% Si as in sample 3 only primary MC, M_6_C and M_3_C carbides are present in the microstructure and no eutectic carbides are found as seen in Fig. 9. The theoretical amount of M6C at different temperatures and at different Si content is shown in Fig. 5. The weight fraction of M_6_C decreases with the addition of Si. Furthermore, when having 3 wt% added Si, two distinct changes of similar kind can be observed according to Thermodynamics. The first occurs at about 950 °C, at which M_7_C_3_, forms and the second is at about 790 °C at which M_23_C_6_ occurs. However, in the sintered microstructure of sample 3, there was no evidence of M_7_C_3_ and M_23_C_6_ carbides.

Mechanical properties of HSS depend mainly on the presence of carbides especially those obtained during solidification and that are known as primary or eutectic carbides or carbides obtained during subsequent heat treatments performed at temperatures high enough to allow either the homogenization of the matrix or the transformation of some primary carbides to secondary carbides. This heat treatment stage is required to produce tailored microstructures that can lead to improved mechanical properties. During subsequent heat treatments of HSS after sintering the M_3_C carbide transforms to other carbide, like M_2_C, MC, M_23_C_6_ and M_7_C_3_. These carbides are called secondary carbides and the MC and M_2_C formation proceed through the dissolution of cementite [15]. The M_2_C carbide is thermodynamically unstable and will decompose into fine M_6_C and MC carbides therefore, the precipitation of M_2_C carbide promotes the refinement of carbide precipitates thus benefiting the mechanical properties of high-speed steels.

Si interacts with C in a repulsive manner resulting in higher carbon activity when these two elements are present in the bulk. This interaction causes carbon to segregate to the grain boundary due to the higher free energy change between the bulk and the grain. This can be best illustrated using the Wagner formalism [16]:

1 $$\ln\;a_{i}^{\gamma }=\ln\;X_{i}^{\gamma }+\sum_{j=1}^{n}\in_{i}^{j}X_{j}$$

where, $X_{i}^{\gamma }$ is the mole fraction of element i in austenite and $\in_{i}^{j}$ is the interaction coefficient between element i and j (Wagner interaction parameter). The interaction coefficient between C and Si is found in Ref. [17] as:

2 $$\epsilon_{C}^{Si}=\frac{7370}{T}+4.84$$

This means more C will segregate towards the grain boundary in the ternary Fe–C–Si system compared to the binary Fe–C system. This is true in the case when the material has been completely melted however in the case when adding Si to HSS powder and then sintering the mixture using liquid phase sintering (solid phase and liquid phase) the repulsive interaction between the slow diffusing Si and the fast-diffusing C will promote the diffusion of C to the interior of the solid particles. This will increase the Si amount at grain boundaries and lower the C amount. The C-rich carbides like MC and M_3_C will form in the cell interior and the low C carbides like the M_7_C_3_ will form along cell boundaries.

Adding 0.5 wt% C and 3 wt% Si to M3:2 HSS (Sample 4) affects the type and morphology, and amount of the carbides present in the microstructure of steel. According to the calculation done using Thermo-calc it is found that with an increase of C, the amount of Mo and C in liquid are increased while other elements like V are decreased. This will favor the solidification of the M_6_C eutectic (seen in Fig. 11) as the solidification proceeds the Mo and W content of the residual liquid is lowered meanwhile the Cr to be enriched this will allow other carbide types such as M_2_C and M_7_C_3_, which are rich in Cr, to precipitate. By examining the microstructure of sample 4 both eutectic M_7_C_3_ and M_6_C carbides are present in the microstructure. The M_7_C_3_ carbide is favored by high Cr and C contents [18]. The microstructure of sample 4 consists of block-like primary M_7_C_3_ and eutectic M_7_C_3_ carbides. The primary M_7_C_3_ carbides are non-closed hollow hexagon-shaped and the eutectic M_7_C_3_ eutectic carbide grow as rods and blades, with the growth direction perpendicular to the image plane, and form a continuous network confined within each eutectic colony.

Three types of eutectic carbide morphologies can be seen in Fig. 11. These are the M_6_C eutectic, having a fishbone morphology, and two M_7_C_3_ eutectic colonies. The first M_7_C_3_ eutectic colony is rod-like consisting of hexagonally shaped rods at the center and becoming coarser as they join with increased distance from the center (Fig. 12). The rod-like morphology is surrounded by blocky primary M_7_C_3_ carbides. The primary M_7_C_3_ has two main features. The first feature is hollows observed in the center of bulky carbides, which are a representative feature of primary M7C3 carbides [19]. Several joints among different parts of the carbides are also frequently observed. This agrees with the findings of reference [20]. The second M_7_C_3_ eutectic colony is blade-like carbides, consisting of rods joining together to form straight blades, which are often described as lamellar (Fig. 11).

Besides the homogeneous nucleation, carbides sometimes nucleate heterogeneously on inclusions like MnS and M (C, N) during solidification [21,22]. From the microstructural examination of sintered samples (Fig. 9, Fig. 10, Fig. 12) none of the MnS and M (C, N) inclusions are seen to exist alone; they are all located on primary and eutectic carbides. According to Aguirre et al. [23] sintering of HSS in the N2 atmosphere leads to the formation of carbonitrides M (C, N).

## 4 Conclusions

Based on the achieved results, the following conclusions can be drawn:

⁃ The addition of 3 wt% Si to HSS suppresses the formation of eutectic carbides.
⁃ The addition of 3 wt% Si and 0.5 wt% C to M3:2 and sintering in N_2_ atmosphere are required for the formation of the M_7_C_3_ carbide, which is preceded by the formation of M_6_C eutectic carbide.

## Data availability statement

The data associated with this study has never been deposited into a publicly available repository. All used data used in this study is included in this manuscript.

## Ethics statements

All authors confirm there is not any financial, personal, or other interests related to the submitted work to disclose.

All authors confirm there are no competing interests to declare.

All authors confirm there is no use of AI nor AI-assisted technologies in the writing process of this work.

## CRediT authorship contribution statement

**Walid Khraisat:** Writing – original draft, Project administration, Methodology, Formal analysis, Conceptualization. **Wisam Abu Jadayil:** Writing – review & editing, Validation, Investigation.

## Declaration of competing interest

We confirm that this work is original and has not been published elsewhere, nor is it currently under consideration for publication elsewhere.

We, all authors, have no conflicts of interest to disclose.
